# MicroRNA Enrichment and Docking-Based Evaluation of Ilomastat Targeting of MMP-2 in Esophageal Squamous Cell Carcinoma: Insights from a South African Cohort

**DOI:** 10.34133/csbj.0097

**Published:** 2026-05-18

**Authors:** Sikhumbuzo Z. Mbatha, Mohammed Alaouna, Botle Damane, Tebogo Marutha, Ottovon B. Dakurah, Zodwa Dlamini

**Affiliations:** ^1^ Department of Surgery, Steve Biko Academic Hospital, University of Pretoria, Hatfield, Pretoria 0028, South Africa.; ^2^ South Africa SAMRC Precision Oncology Research Unit (PORU), DSI/NRF SARChI Chair in Precision Oncology and Cancer Prevention (POCP), Pan African Cancer Research Institute (PACRI), University of Pretoria, Hatfield, Pretoria 0028, South Africa.; ^3^ Centre for Bioinformatics and Computational Biology, Faculty of Science, Stellenbosch University, Stellenbosch, South Africa.; ^4^ Wolfson Wohl Cancer Research Centre, School of Cancer Sciences, University of Glasgow, Glasgow G61 1QH, UK.

## Abstract

**Introduction:** MicroRNAs (miRNAs) play central roles in cancer pathogenesis by modulating oncogenic and tumor-suppressive pathways. The microRNA-29 (miR-29) family, known to inhibit matrix metalloproteinase-2 (MMP-2), represents a key regulatory axis in tumor invasion. This study analyzed miRNA pathway enrichment derived from tumor-normal mRNA differential expression in esophageal squamous cell carcinoma (ESCC) patients and investigated whether Ilomastat, a known MMP inhibitor, could plausibly bind the catalytic site of MMP-2. **Methods:** Thirty-eight patients with suspected esophageal cancer were recruited from an Academic Hospital in 2024. Twenty-nine ESCC patients were finally included. Paired tumor/adjacent-normal tissues were collected using endoscopic biopsies. Total RNA was extracted, sequenced, and bioinformatics conducted using the nf-core/rnaseq pipeline, with pathway enrichment analysis completed using g:Profiler2. Additional computational docking studies were performed to assess whether Ilomastat tautomers could plausibly bind the catalytic site of MMP-2. **Results and Discussion:** Pathway enrichment analysis identified several miRNAs implicated in ESCC pathogenesis, including the miR-29 family. Transcriptome analysis revealed overexpression of LINC00392 and FABP4 (fatty acid-binding protein 4) among the differentially expressed genes. FABP4 drives lipid metabolic reprogramming and tumor microenvironments that support cancer cell proliferation and metastatic potential. Computational docking suggested that the neutral tautomer of Ilomastat favorably occupies the catalytic site of MMP-2, providing a hypothesis-generating structural rationale for downstream pharmacologic inhibition of MMP-2. **Conclusion:** This study integrates pathway-based miRNA enrichment and computational docking to highlight the miR-29–MMP-2 axis as a potential regulatory pathway in ESCC. Collectively, our findings provide hypothesis-generating support for miRNA-guided MMP-2 inhibitor design that bridges transcriptomic discovery with pharmacologic modeling.

## Graphical Abstract

**Figure:**
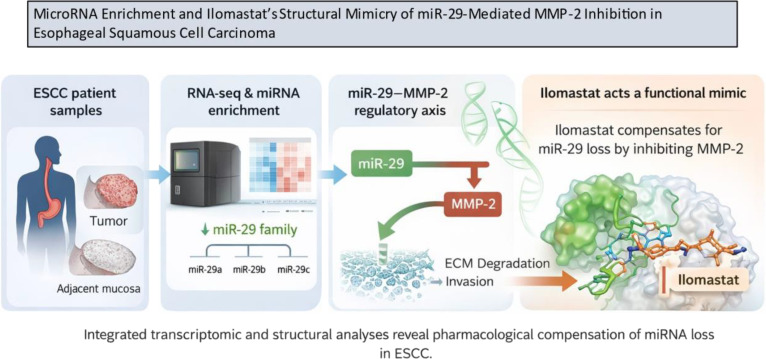

## Introduction

MicroRNAs (miRNAs) are a class of noncoding RNAs that are 18 to 22 nucleotides long. They play critical roles in numerous aspects of cell biology, including cell proliferation, differentiation, and tumorigenesis. Additionally, these miRNAs remain extremely stable in bodily fluids and tissues and have proven to be excellent potential cancer biomarkers [1]. For instance, analysis of ESCC miRNA datasets by Miyoshi *et al.* yielded 8 candidate miRNAs, including miR-103, miR-106b, miR-151, miR-17, miR-181a, miR-21, miR-25, and miR-93, whose serum overexpression produced an “8-miR” liquid biopsy signature. This diagnostic tool surpassed the specificity of SCC-associated antigens, providing strong evidence for multi-miRNA panel screening approaches [2].

miRNAs were recognized as a large family of short RNAs found in higher eukaryotes in the year 2001 [3]. Most arise through a strict canonical pathway; however, alternative, noncanonical biogenetic routes have also been described [4].

The dysregulation of miRNAs has been increasingly apparent in most, if not all, malignancies. Many of these miRNAs either promote or suppress cancer by blocking the expression of tumor suppressors or oncogenes. Oncogenic miRNAs (oncomiRs) are overexpressed in malignancies, whereas tumor-suppressive miRNAs are underexpressed [5]. Numerous studies have reported that miRNAs affect cancer through various mechanisms, including tumor angiogenesis, cell differentiation, proliferation, and metabolism. A single miRNA can potentially target multiple mRNAs. Additionally, because miRNAs are relatively stable in physiological fluids, liquid biopsy approaches have emerged as minimally invasive tools for early cancer detection and longitudinal disease monitoring [6]. Moreover, a better understanding of miRNA function in cancer biology has important implications for the development of novel therapeutic strategies [7].

Several cellular and molecular regulators, including transcription factors (TFs), signaling pathways, and miRNAs, influence the formation and progression of esophageal squamous cell carcinoma (ESCC), resulting in significant genetic and molecular heterogeneity in ESCC. Understanding and quantifying these factors enhances the diagnostic, prognostic, and predictive precision in ESCC, facilitating the design of personalized therapeutic approaches [8]. Interestingly, studies have shown that, besides the miRNA effect, the prognosis and progression of esophageal cancer can be strongly influenced by various other factors that affect the tumor microenvironment, including the types of cells surrounding the tumor cells, mitochondrial energy metabolism (MEM), and the microbiological organisms prevalent in tumor tissues [9,10]. For example, *Fusobacterium nucleatum* plays an important role in ESCC progression and prognosis. It is closely related to the pathologic and clinical stages of ESCC and is found more commonly in metastatic ESCC than in nonmetastatic ESCC. Patients with *F. nucleatum*-positive ESCC had shorter cancer-specific survival and higher cancer-specific mortality. Furthermore, a number of studies have convincingly proven that ESCC tissue exhibits radically distinct microbiome than surrounding normal tissues [9].

Given their regulatory versatility, miRNAs play a central role in ESCC pathogenesis, functioning as either tumor suppressors or oncogenes, depending on the cellular context. For instance, MiR-125b functions as a tumor suppressor in many solid tumors (such as prostate cancer), but functions as an oncomiR in most hematological malignancies. It was discovered that miR-125b is overexpressed in prostate cancer samples and promotes androgen-independent growth of prostate cancer cells. In breast cancer, miR-125b suppresses the oncoproteins Mucin 1 (MUC1), Erb-B2 Receptor Tyrosine Kinase 2 (ERBB2), and ERBB3, which hinders the growth of breast cancer cells and increases their susceptibility to genotoxic anticancer drugs [11]. Additionally, it was shown in an *in vivo* mouse model of acute myocardial infarction that intravenous administration of miR-125b mimic conferred cardio-protection during ischemia and significantly decreased myocardial infarct size, by targeting and down-regulating the mRNA expression of Ccna2 and Eef2k [12]. In esophageal cancer, it exhibits tumor-suppressive properties [13]. Additional research has shown that certain malignancies, such as gastric cancer, are characterized by ethnic and geographic variables that shape both transcriptional and posttranscriptional miRNA expression patterns [14].

ESCC is one the most fatal malignancies globally and is characterized by delayed presentation with advanced disease due to lack of distinct early symptoms [15]. Metastasis is the hallmark of cancer responsible for the majority of cancer-related fatalities [16]. Various compounds have been linked to cancer progression including zinc-containing endopeptidases known as matrix metalloproteinases (MMPs), which are primarily made up of MMP-2 and MMP-9. They are involved in several biological processes, including cell proliferation, migration, differentiation, apoptosis, and angiogenesis. They also significantly contribute to the breakdown of extracellular matrix (ECM) components. Additionally, MMP-2 has been shown to be overexpressed in a number of solid tumors, including ESCC, where it is associated with metastases and the advancement of the malignancy [17].

Current treatment modalities for ESCC include a combination of radiotherapy, chemotherapy, and surgery [18,19]. More recently, immune checkpoint inhibitors and targeted therapies are increasingly playing a significant role as therapeutic agents demonstrating improved overall and disease-free survival rates in advanced or residual disease following tri-modality treatment [20]. Advanced and metastatic disease is a major factor influencing treatment outcomes. MMP-2 overexpression in ESCC has been associated with cell migration and metastases [21], and MMP-2 inhibition may offer therapeutic potential for ESCC as it has been shown to attenuate the cell migration, invasion, and metastatic potential in oral squamous cell carcinoma cell lines [22].

Kim *et al.* examined the highly metastatic human fibrosarcoma cell line HT1080 to determine whether miR-29s could control cancer cell invasion by blocking MMP-2. They showed that the 3′-untranslated region of MMP-2 was specifically targeted by miR-29s, and that the expression of miR29s caused MMP-2 to be down-regulated and its activity to decrease in cancer cells [23]. Using both *in vitro* and *in vivo* techniques, Fang *et al.* [24] showed that miR-29b directly targets MMP-2 in hepatocellular carcinoma cell lines and that by inhibiting MMP-2, miR-29b suppresses angiogenesis, invasion, and metastasis. MMP-2 suppression with Ilomastat has also been demonstrated to provide considerable cardiocytoprotection in rats. Ilomastat, whether delivered before ischemia or before reperfusion, was able to considerably reduce infarct size [25].

Collectively, these insights underscore the pivotal role of miRNAs in ESCC biology and highlight their potential as diagnostic biomarkers and therapeutic targets for precision oncology. This study aimed to characterize miRNA pathway enrichment inferred from mRNA-level differential expression in South African ESCC tissues and to investigate whether Ilomastat, a known MMP inhibitor, could plausibly bind the catalytic site of MMP-2, thereby providing a hypothesis-generating rationale for downstream pharmacologic targeting of MMP-2 within the miR-29/MMP-2 axis.

## Methods and Materials

### Study setting, sample collection, and sample processing

This observational study was conducted from February to December 2024. Thirty-eight patients (*n* = 38) with a clinical presentation of esophageal cancer were seen at the Department of Surgery, Steve Biko Academic Hospital, a level 4 referral center affiliated with the University of Pretoria, Pretoria, South Africa. All patients were interviewed and provided written informed consent prior to their inclusion in the study. Each participant underwent upper endoscopy with paired tumor and adjacent normal tissue biopsy and human immunodeficiency virus (HIV) testing (or verification of existing antiretroviral therapy status). Ethical approval was obtained from the University of Pretoria Research Ethics Committee (Ethics Reference No. 296/2021) and the Gauteng Health Department (NHRD Ref. No. GP_202107_062). The inclusion criteria for the study were confirmed ESCC and a known verifiable HIV status. Only 29 patients met the inclusion criteria. Tumor differentiation status was abstracted from the diagnostic pathology reports and categorized as well-, moderately, or poorly differentiated squamous cell carcinoma, with a small subset reported only as “invasive carcinoma” without formal grading.

### Tissue collection

Fresh tissue-paired tumor and adjacent normal biopsies were obtained via endoscopy. Normal adjacent tissues were collected more than 2 cm from the gross margin of the tumor. These fresh tissues were placed in microcentrifuge tubes containing RNAprotect reagent (Qiagen), snap-frozen in liquid nitrogen within 1 h of collection and stored at −80°C until RNA extraction. Further samples were sent for histological confirmation of ESCC and subsequently for human papillomavirus (HPV) genotyping.

### RNA sequencing

RNA was extracted from fresh tissue samples using the Quick-RNA Miniprep Plus Kit according to the manufacturer’s instructions (Zymo Research, Irvine, CA, USA). RNA concentration and integrity were verified using a Qubit 4 Fluorometer and an RNA IQ Assay Kit (Table S1) (Thermo Fisher Scientific, Waltham, MA, USA). cDNA synthesis was performed using the NEBNext Single Cell/Low Input cDNA Synthesis Module (NEB #E6421) (New England Biolabs, USA), and double-stranded DNA (dsDNA) quality was confirmed with a high-sensitivity Qubit fluorometer (Invitrogen, Carlsbad, CA, USA) using dsDNA assay (Invitrogen, Carlsbad, CA, USA, cat: Q32854).

Sequencing libraries were constructed using the Illumina DNA Prep Kit (Illumina, San Diego, USA, Cat. no. 20060059) and evaluated for quality and fragment distribution using the Agilent TapeStation System (Agilent Technologies, Santa Clara, USA). Libraries were sequenced in paired-end mode (2 × 150 bp) on the Element AVITI platform according to the manufacturer’s specifications (Element Biosciences, San Diego, USA).

### Bioinformatics analysis

Differential abundance analysis comparing tumor and matched normal tissues was performed using the publicly available nf-core/rnaseq pipeline (https://github.com/nf-core/rnaseq), a standardized workflow for RNA sequencing (RNA-seq) quality control, alignment, and quantification. The pipeline accepts FASTQ files and metadata sheets, performs quality assessment and alignment, and generates an annotated gene expression matrix.

**Fig. 1.**
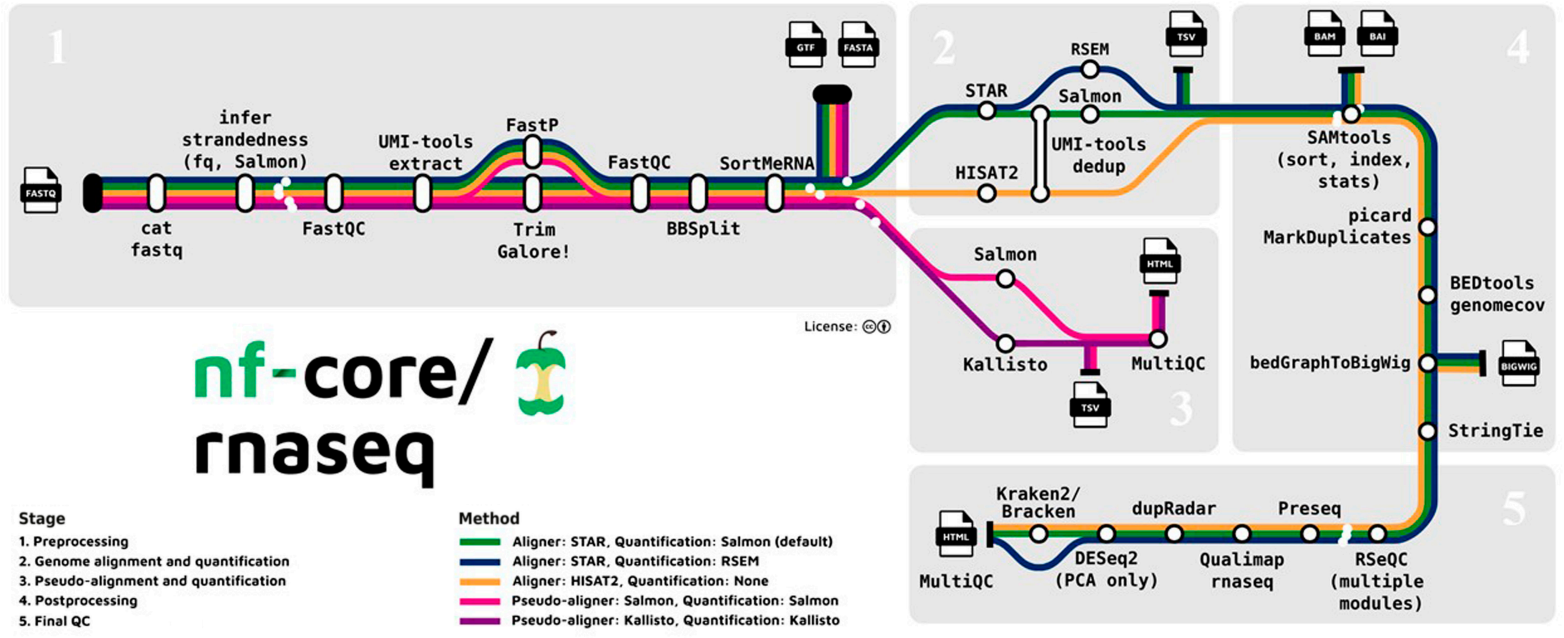
Overview of the nf-core/rnaseq pipeline used for RNA-seq data processing (adapted from https://github.com/nf-core/rnaseq).

Data were processed using nf-core/rnaseq v3.18.0 (doi:10.5281/zenodo.1400710) of the nf-core workflow collection [26] within reproducible Bioconda [27] and Biocontainers [28] environments. The analysis workflow is illustrated in Fig. 1.

The pipeline was executed using Nextflow v24.10.3 [29] with the following command:

*nextflow run nf-core/differentialabundance -profile rnaseq,apptainer --input samplesheet.csv --contrasts contrasts.bak.tsv --matrix rsem.merged.gene_counts.tsv --gtf Homo_sapiens.GRCh38.113.gtf.gz --outdir DE_MBATHA9 -c nextflow.config --gsea_run true --gene_sets_files c2.all.v2024.1.Hs.symbols.gmt --gprofiler2_run true --gprofiler2_organism hsapiens --deseq2_alpha 0.05*

### Differential analysis

Differential gene expression between tumor and adjacent normal tissues was analyzed using the DESeq2 R package. *P* values were adjusted for multiple testing using the Benjamini–Hochberg false discovery rate (FDR) method to minimize false positives.

Genes were considered differentially expressed if they met both criteria.

• The adjusted *P* value was equal to or lower than 0.05
• Absolute log-fold change (|log₂FC|) ≥ 1.0.

The input was a matrix of 78,932 genes for 58 samples, which was reduced to 49,606 genes after filtering for low abundance.

In exploratory analyses, an extended DESeq2 design, including clinical covariates (HIV status, age, sex, smoking, alcohol use, and tumor location), was also evaluated. This covariate-adjusted model yielded a more permissive DEG set with an increased number of significant genes relative to the simpler design, likely reflecting the limited cohort size and the potential for overfitting. To retain a conservative, stringent framework for downstream enrichment analyses, the primary results presented here are based on a simpler tumor-versus-normal model, with covariate effects examined at the level of principal components.

Although more advanced approaches for modeling hidden or complex covariate structures in RNA-seq data, such as surrogate variable analysis and pseudo-variable augmentation, are available, the present study relied on a standard DESeq2 design with covariate-expanded exploratory models and PCA-based inspection of covariate effects, which we judged appropriate given the modest cohort size and hypothesis-generating scope of the analysis.

#### g:Profiler for pathway enrichment analysis

The differentially expressed genes (DEGs) were subsequently analyzed for pathway enrichment using g:Profiler2 (http://biit.cs.ut.ee/gprofiler/gost) within the R environment, with parameters set as depicted in (Table 1) [30]. Pathway enrichment significance was defined at an adjusted *P* value ≤ 0.05, using the gSCS (Gene Set Compression Scheme) multiple-testing correction method. The differential fraction for each pathway was computed as the ratio of significant genes to the total annotated genes. The differential fraction is the number of differential genes in a pathway divided by the size of that pathway, that is, the number of genes annotated for the pathway.

**Table 1.** Parameters of the g:Profiler pathway analysis

Parameters used for g:Profiler2	
Parameter	Value
Run	TRUE
Organism	hsapiens
Significant	TRUE
measure_underrepresentation	FALSE
correction_method	gSCS
Evcodes	FALSE
max_qval	0.05
background_file	auto
domain_scope	annotated
min_diff	1
palette_name	Blues

Pathway enrichment analysis (PEA) is a computational approach that identifies biological processes that are disproportionately represented in a given gene list [31]. The g:Profiler2 suite integrates Gene Ontology (GO), Kyoto Encyclopedia of Genes and Genomes (KEGG), Reactome, and miRNA pathway databases to determine the enriched biological functions and molecular networks associated with DEGs in ESCC [32].

### Computational docking analysis

Computational docking analysis was performed to evaluate whether Ilomastat could functionally mimic miR-29-mediated repression of MMP-2. The human MMP-2 catalytic domain (Protein Data Bank [PDB] ID: 1CK7) was prepared using the Schrödinger Protein Preparation Wizard, maintaining the tetrahedral coordination of Zn^2+^ by His403, His407, His413, and Glu404. LigPrep generated 2 protonation states of Ilomastat (neutral and anionic), which were docked using Glide SP, followed by XP refinement.

Hydroxamate-based MMP inhibitors typically have p*K*_a_ values in the ~8.5 to 9.5 range, indicating that the neutral (protonated) form remains substantially populated at physiological pH and is even more favored in a mildly acidic tumor microenvironment. Therefore, both neutral and anionic Ilomastat tautomers were docked. The stronger binding observed for the neutral form in our results reflects not the assumed dominance of this tautomer but its intrinsically more favorable Zn^2+^ coordination geometry.

A receptor grid was centered on the zinc-binding region with dimensions sufficient to encompass the catalytic cleft while restricting sampling to the active-site pocket. Docking was performed using a rigid receptor and full-ligand conformational sampling. The top-ranked poses were subsequently rescored using the MM-GBSA (VSGB 2.1) model, permitting the local relaxation of residues within 5 Å of the ligand.

#### Protein preparation

The human MMP-2 catalytic domain (PDB ID: 1CK7) was imported into Schrödinger Maestro (version 2025-3) and prepared using the Protein Preparation Wizard.

All water molecules beyond 5 Å from the cocrystallized inhibitor were deleted. Missing side chains were added and optimized under the OPLS4 force field, maintaining the tetrahedral geometry of the catalytic zinc center coordinated to His 403, His 407, His 413, and Glu 404.

#### Ligand preparation

The structure of ilomastat (CID 132519) was obtained from PubChem and processed using LigPrep to generate 2 protonation states.

• Ilomastat-1 (neutral)-hydroxamic group is protonated.
• Ilomastat-2 (anionic)-deprotonated hydroxamate oxygen.

The ligands were geometrically optimized using OPLS4 with default convergence.

#### Grid generation and docking

The receptor grid was centered on the zinc-binding motif (His 403–His 407–His 413–Glu 404), extending 20 Å^3^. Docking was performed using Glide SP, followed by Glide XP refinement, and all rotatable bonds were sampled. The top-ranked poses were further rescored using Prime MM-GBSA (VSGB 2.1 solvent model), with residues within 5 Å of the ligand permitted to relax.

#### Visualization and interaction mapping

Docking poses were analyzed in the Maestro Workspace and visualized as ball-and-stick models with ribbon secondary structures. Electrostatic potential surfaces (–0.3 → 0.0 e/Å^2^) were generated, and 2D interaction diagrams were rendered with a ligand interaction diagram.

## Results

### Demographic data

The overall characteristics of the 29 patients included in this analysis are summarized in Table 2, stratified by HIV status. The cohort was predominantly female (58.6%) and of African descent. Most patients were older than 50 years (89.7%). HIV infection was present in 51.7% of the patients. HPV was detected in 72.4% of patients, with HPV16 being the most frequently detected type (58.6%) and HPV18 being observed in 27.6% of patients. Comorbidities were reported in 34.5% of patients, and a family history of cancer was rare (10.3%). Alcohol use and smoking were reported by 39.3% and 28.6% of patients, respectively, with 29.6% of patients reporting both alcohol use and smoking. However, overall, there was a statistically significant association between HIV and ESCC.

**Table 2.** Fisher’s exact test analysis of demographic and clinical factors by HIV status (*n* = 29)

Characteristic	HIV-negative (*n* = 14)	HIV-positive (*n* = 15)	Total (*n* = 29)	*P* value
Gender				0.26
Male	4 (29.0%)	8 (53.0%)	12 (41.0%)	
Female	10 (71.0%)	7 (47.0%)	17 (59.0%)	
Age category				0.22
≤50	0 (0.0%)	3 (20.0%)	3 (10.3%)	
>50	14 (100.0%)	12 (80.0%)	26 (89.7%)	
HPV status				0.68
Negative	3 (21.4%)	5 (33.3%)	8 (27.6%)	
Positive	11 (78.6%)	10 (66.7%)	21 (72.4%)	
HPV16				0.078
HPV16 negative	0 (0.0%)	4 (26.7%)	4 (13.8%)	
HPV16 positive	11 (78.6%)	6 (40.0%)	17 (58.6%)	
HPV not detected	3 (21.4%)	5 (33.3%)	8 (27.6%)	
HPV18				0.013
HPV18 negative	10 (71.4%)	3 (20.0%)	13 (44.8%)	
HPV18 positive	1 (7.1%)	7 (46.7%)	8 (27.6%)	
HPV not detected	3 (21.4%)	5 (33.3%)	8 (27.6%)	
Comorbidity				0.021
No	6 (42.9%)	13 (86.7%)	19 (65.5%)	
Yes	8 (57.1%)	2 (13.3%)	10 (34.5%)	
Family history of cancer				1.00
No	13 (92.9%)	13 (86.7%)	26 (89.7%)	
Yes	1 (7.1%)	2 (13.3%)	3 (10.3%)	
Alcohol use				0.024
No	11 (84.6%)	6 (40.0%)	17 (60.7%)	
Yes	2 (15.4%)	9 (60.0%)	11 (39.3%)	
Smoking				0.038
No	12 (92.3%)	8 (53.3%)	20 (71.4%)	
Yes	1 (7.7%)	7 (46.7%)	8 (28.6%)	
Alcohol and smoking				0.043
No	11 (91.7%)	8 (53.3%)	19 (70.4%)	
Yes	1 (8.3%)	7 (46.7%)	8 (29.6%)	
Both HPV16 and HPV18 positive				0.31
No	10 (90.9%)	7 (70.0%)	17 (81.0%)	
Yes	1 (9.1%)	3 (30.0%)	4 (19.0%)	

Histopathology showed that most tumors were moderately differentiated ESCC, with smaller subsets of well-differentiated, poorly differentiated, and ungraded “invasive carcinoma” cases. In exploratory checks, the sample clustering and principal component structure were not obviously driven by differentiation status; therefore, all 29 graded and ungraded tumors were retained in the primary tumor versus normal analyses.

### Gene differential expression

**Fig. 2.**
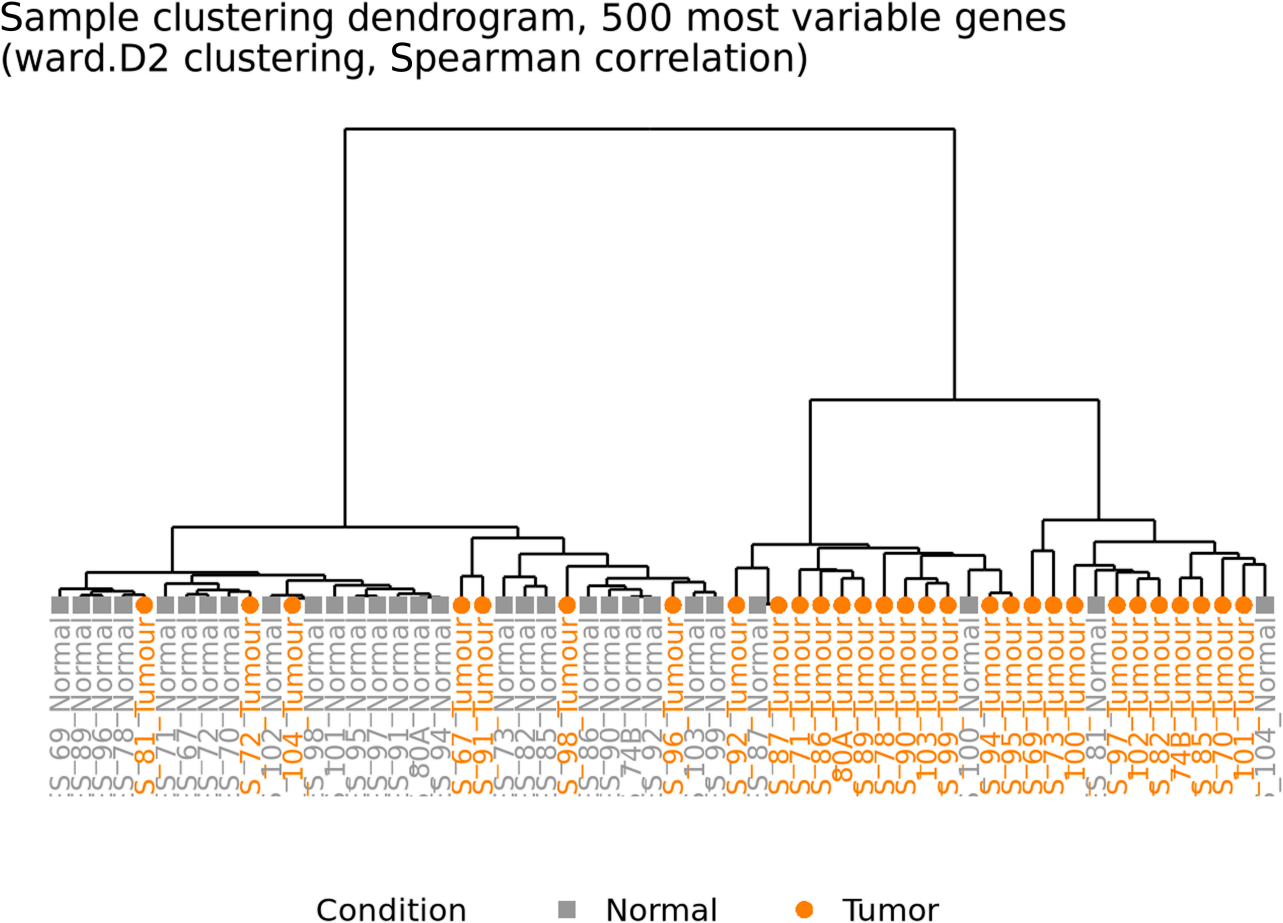
Sample hierarchical clustering dendrogram: It shows 2 distinct major groupings of the main branches, with the group on the right dominated by tumor (orange) and the left dominated by normal samples (gray), underscoring a robust biological clustering and differentiation between tumor and normal tissues. Despite a few intermixed samples on either side, the emerging picture suggests a substantial divergence of the global transcriptional profiles of tumor tissues from those of normal mucosal tissues. The short first-level substructure heights in normal samples demonstrate limited variability, whereas the larger first-level substructure of the tumor highlights tumor heterogeneity. This robust clustering based on the tissue type (tumor *vs.* normal) supports the notion of limited batch effect in our analysis

The nf-core/differential abundance pipeline was used to assess the differential gene expression between the overall tumor samples and the corresponding adjacent normal tissues. An input matrix of 78,932 genes from 58 samples was used. After filtering low-abundance genes, 49,606 genes remained for further analysis. Variance-based gene filtering was performed, and the top 500 most variable genes across all samples were selected for the unsupervised clustering. Hierarchical clustering of genes was performed. Distances between genes were estimated based on Spearman correlation, which were then used to produce a clustering via the ward.D2 method with hclust() in R. Figure 2 depicts the sample dendrogram.

### Principal component analysis

**Fig. 3.**
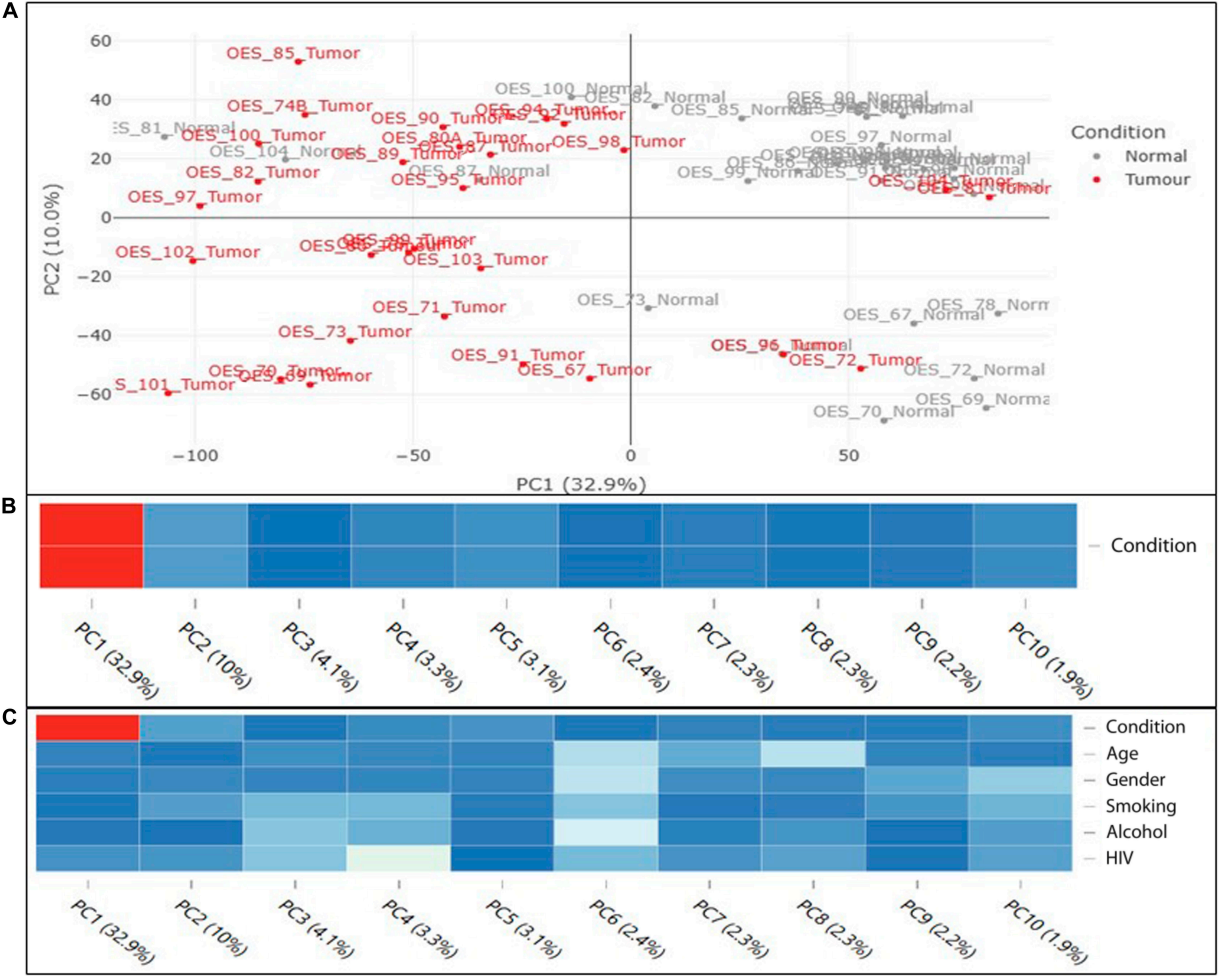
(A) Plots depicting principal component analysis: variance stabilized for condition (tumor *vs.* normal). (B) *P* values of PCA: The variable “condition” showed an association with PC1 (32.9%) (*P* = 0.00). (C) When other covariates were included in the model, most differentially expressed genes were still influenced predominantly by tumor versus normal conditions, with other factors showing some effect but to a much lesser extent.

Principal component analysis (PCA) was conducted based on the 500 most variable genes. Each component was annotated with its percent contribution to the variance, as depicted in Fig. 3. For the variance-stabilized matrix, an analysis of variance test was used to determine the associations between continuous principal components and categorical covariates (including the variable of interest).

The PCA demonstrated a clear differentiation between normal and tumor tissues, with most of the separation evident at PC1, which is the premier axis differentiation. Most tumor samples were distributed on the left, and normal samples clustered mostly to the right of PC1. This clear distinction displays a clear biological signal with a consistent molecular signature distinguishing tumors from normal tissues. Tumor samples showed a wide distribution across PC2, indicative of tumor heterogeneity, while normal samples were mostly clustered together, in keeping with high tissue consistency and minimal biological variance. The robust distinction between tumor and normal tissue samples suggests a tumor-specific expression profile. The other variables showed lower percentage associations; for instance, the variable “age” showed an association with PC6 (2.4%) (*P* = 0.01). The variable “gender” was associated with PC6 (2.4%) (*P* = 0.01). The variable “smoking” was associated with PC3 (4.1%) (*P* = 0.07). The variable “alcohol” was associated with PC3 (4.1%) (*P* = 0.04). The variable “HIV” was associated with PC3 (4.1%) (*P* = 0.04).

**Fig. 4.**
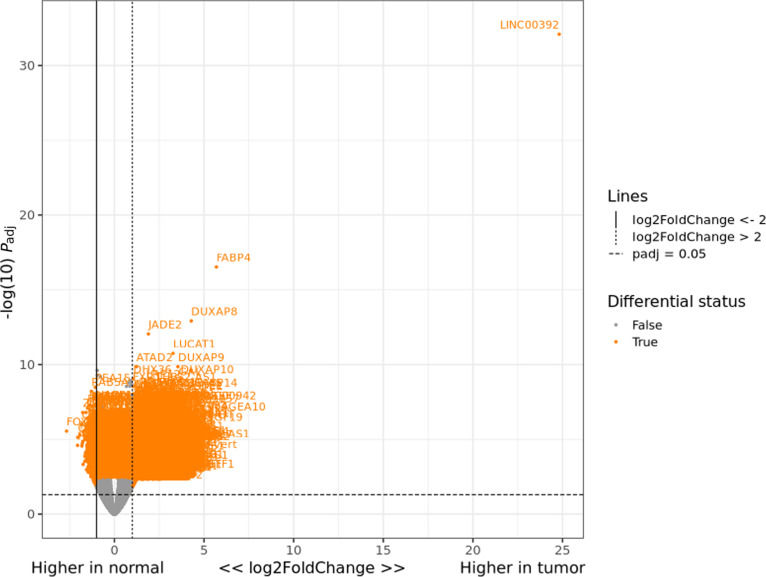
Volcano plot showing the differential gene expression profiles between tumor and normal tissues. Each point represents an individual gene, plotted by log2 fold change (*x*-axis) versus statistical significance (−log10 adjusted *P* value, *y*-axis). Genes that met the criteria for significant differential expression (adjusted *P* value < 0.05 and |log2 fold change| > 2) are labeled and colored orange, whereas nonsignificant genes are shown in gray. The most markedly up-regulated genes in tumor tissue, LINC00392 and FABP4, are observed as extreme outliers on the far right, indicating pronounced elevation in tumor tissues.

**Fig. 5.**
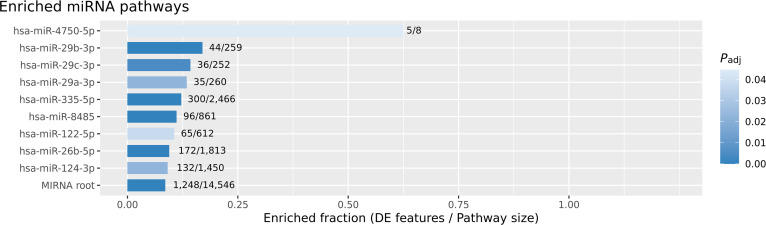
Enriched miRNA pathways identified by pathway analysis of differentially expressed genes. The bar plot displays the fraction of pathway genes found among the differentially expressed features for each enriched miRNA pathway, with the denominator indicating the total pathway size. Pathways were ordered by enrichment, and the bar color reflects statistical significance (adjusted *P* value, *P*_adj_). The miRNA root indicated that the miRNA pathway was significantly affected and enriched in this sample collection. The miR-29 family members (hsa-miR-29b-3p, hsa-miR-29c-3p, and hsa-miR-29a-3p) are prominently enriched, alongside hsa-miR-335-5p, hsa-miR-8485, hsa-miR-122-5p, hsa-miR-26b-5p, and hsa-miR-124-3p. Although hsa-miR-4750-5p revealed a highly enriched fraction (0.625), the pathway was miniscule, hence lower significance. This analysis highlights the key miRNA regulatory networks implicated in ESCC, with enrichment fractions indicating the proportional representation of differentially expressed targets within each pathway. Several other pathways with lowly enriched fractions or large background sizes were observed but contributed minimally to the overall biological interpretation.

A volcano plot illustrating the DEGs is presented in Fig. 4. Among the DEGs, FABP4 and LINC000392 appeared as marked outliers, showing strong overexpression in tumor tissues (Fig. 4). Elevated FABP4 expression has been associated with lipid-metabolic reprogramming and tumor-microenvironment remodeling, which is consistent with the extreme fold change observed in this dataset. The DEGs were subsequently subjected to pathway and miRNA enrichment analyses (Fig. 5)

### MiRNA PEA

The DEGs identified through differential abundance assessment were subjected to PEA using the g:Profiler software (http://biit.cs.ut.ee/gprofiler/gost). Working with ranked or sorted lists of genes is a key aspect of g:Profiler [32]. Several enriched pathways were identified, including enriched micro-RNAs (Fig. 5), CORUM pathways, KEGG pathways, GO: BP/GO:CC/GO:MF, HPA Pathway, REAC, and WP pathways. Table 3 shows the enriched miRNAs and their adjusted *P* values.

**Table 3.** Enriched microRNAs and related pathways with their adjusted *P* values derived using GP profiler-2

Pathway name	Pathway code	DE genes	Pathway size	Fraction DE	*P*_adj_
MIRNA root	MIRNA:000000	1,248	14,546	0.0858	0
hsa-miR-335-5p	MIRNA:hsa-miR-335-5p	300	2,466	0.1217	0
hsa-miR-29b-3p	MIRNA:hsa-miR-29b-3p	44	259	0.1699	0
hsa-miR-8485	MIRNA:hsa-miR-8485	96	861	0.1115	1e-04
hsa-miR-26b-5p	MIRNA:hsa-miR-26b-5p	172	1,813	0.0949	1e-04
hsa-miR-29c-3p	MIRNA:hsa-miR-29c-3p	36	252	0.1429	0.0045
hsa-miR-29a-3p	MIRNA:hsa-miR-29a-3p	35	260	0.1346	0.0222
hsa-miR-124-3p	MIRNA:hsa-miR-124-3p	132	1,450	0.091	0.0223
hsa-miR-122-5p	MIRNA:hsa-miR-122-5p	65	612	0.1062	0.0378
hsa-miR-4750-5p	MIRNA:hsa-miR-4750-5p	5	8	0.625	0.0446

### Computational docking analysis of the miR-29–MMP-2 axis using Ilomastat as a putative MMP-2 inhibitor

Docking and MM-GBSA rescoring revealed a clear distinction in the binding affinity between the 2 Ilomastat protonation states. The neutral tautomer (Ilomastat-1) exhibited the most favorable energetics, with Glide SP = –4.244 kcal·mol^−1^, Glide XP = –4.237 kcal·mol^−1^, and Δ*G*_bind_ = –30.02 kcal·mol^−1^ (Table 4). In contrast, the anionic tautomer (Ilomastat-2) demonstrated weaker binding (XP = –2.804 kcal·mol^−1^; Δ*G*_bind_ = –19.85 kcal·mol^−1^).

**Table 4.** Docking energetics: The neutral Ilomastat tautomer (Ilomastat-1) exhibited the most favorable docking and binding energies, consistent across the SP, XP, and MM-GBSA scoring functions

Ligand	Glide SP (kcal·mol^−1^)	Glide XP (kcal·mol^−1^)	Emodel	Δ*G*_bind_ (MM-GBSA, kcal·mol^−1^)	Prime energy
Ilomastat-1 (neutral)	–4.244	–4.237	–48.629	–30.02	–24,279.55
Ilomastat-2 (anionic)	–0.455	–2.804	–28.751	–19.85	–24,286.95

These values indicate that the protonation state markedly influences the stability of the inhibitor within the catalytic pocket. The stronger binding of Ilomastat-1 is consistent with the optimal positioning of its hydroxamate moiety and enhanced stabilization through surrounding polar and hydrophobic interactions.

### Binding mode of neutral Ilomastat (Ilomastat-1)

**Fig. 6.**
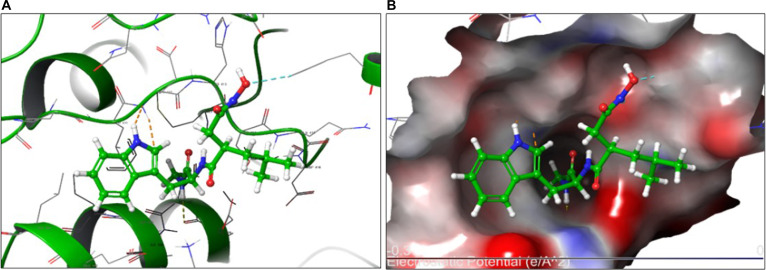
Ilomastat-1: Three-dimensional binding interactions and electrostatic surface analysis of Ilomastat-1 in the MMP-2 active site. (A) Binding pose of Ilomastat-1 illustrating key polar interactions with His413, Ser414, Gln415, Asp416, Asp436, and Lys439, together with hydrophobic contacts involving Leu411 and Leu444. (B) Electrostatic potential surface of Ilomastat-1 within the catalytic pocket of MMP-2 (–0.3 to 0 e/Å²), highlighting the distribution of charge and inhibitor positioning that supports optimal binding affinity.

The top-ranked pose of Ilomastat-1 positioned the hydroxamate group deep within the catalytic cleft, orienting its oxygen atoms toward the zinc-coordinated region of the enzyme. This binding mode was associated with a stabilizing polar interaction network within the active site, together with hydrophobic contacts involving Leu411, Met421, and Leu444 (Fig. 6A). Electrostatic surface mapping further revealed strong complementarity between the negatively polarized hydroxamate region of Ilomastat and the positively charged environment surrounding Zn^2+^ (Fig. 6B), consistent with the favorable MM-GBSA energy observed.

**Fig. 7.**
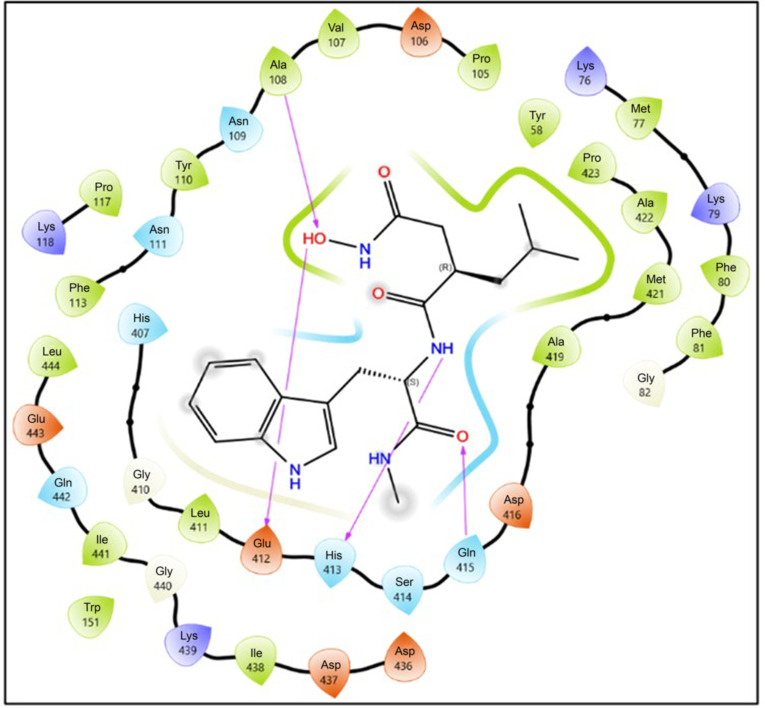
Two-dimensional ligand interaction diagram of the top-ranked neutral Ilomastat (Ilomastat-1) pose within the MMP-2 catalytic pocket. Purple arrows indicate hydrogen bonds. In this representation, the clearest direct hydrogen-bond contacts are observed with Ala108, Glu412, His413, and Gln415, while nearby residues including Ser414, Asp416, Met421, Ala422, Pro423, Leu411, and Leu444 define the surrounding pocket environment. The figure illustrates the local interaction network supporting stable accommodation of the neutral Ilomastat tautomer within the catalytic region of MMP-2.

The revised 2D interaction map (Fig. 7) further clarifies the local contact pattern supporting this pose. In this representation, the clearest direct hydrogen-bond interactions are observed with Ala108, Glu412, His413, and Gln415, while nearby residues including Ser414, Asp416, Met421, Ala422, Pro423, Leu411, and Leu444 define the surrounding catalytic-pocket environment. Purple arrows in the figure denote hydrogen bonds. Together, these interactions support stable accommodation of the neutral Ilomastat tautomer within the catalytic region of MMP-2 and are consistent with its more favorable predicted binding relative to the anionic tautomer.

The binding pose of neutral Ilomastat also aligns with key features reported for classical hydroxamate-based MMP-2 inhibitors. In inhibitor-bound MMP-2 complexes, effective ligands typically coordinate the catalytic Zn^2+^ ion through the hydroxamate group while extending into the hydrophobic S1′ pocket. In our docking model, Ilomastat-1 reproduced these general binding characteristics, with the hydroxamate moiety oriented toward the zinc-binding region and the P1′ side chain projecting toward the Leu411/Met421/Leu444 pocket. These observations indicate that Ilomastat-1 engages MMP-2 through a binding mode consistent with established hydroxamate-based inhibitor architecture, supporting the plausibility of the predicted pose while remaining hypothesis-generating.

### Binding mode of anionic Ilomastat (Ilomastat-2)

**Fig. 8.**
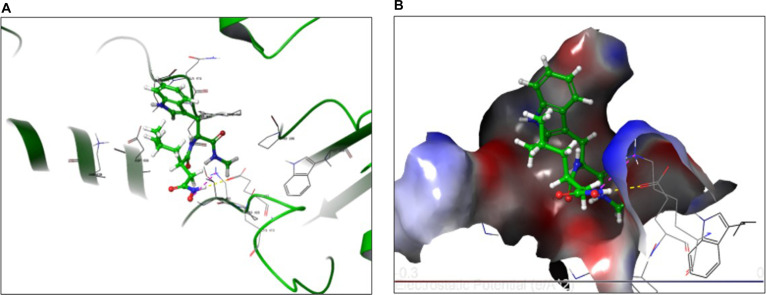
Ilomastat-2: Structural and electrostatic analysis of Ilomastat-2 interactions with MMP-2. (A) Three-dimensional binding pose of Ilomastat-2 within the MMP-2 active site, showing hydrogen bonds with Asp416, Ser414, Gln415, Lys470, and Glu467. (B) Electrostatic potential surface representation of Ilomastat-2 in the catalytic pocket, demonstrating areas of partial charge repulsion near Glu467, which may influence inhibitor binding affinity and orientation.

Although Ilomastat-2 bound within the same catalytic region, its deprotonated hydroxamate group adopted a rotated orientation that reduced complementarity with zinc-associated residues (Fig. 8A). The dominant interactions shifted toward Lys470 and Glu467**,** supported by weaker contacts with Asp416, Ser414, and Gln415.

**Fig. 9.**
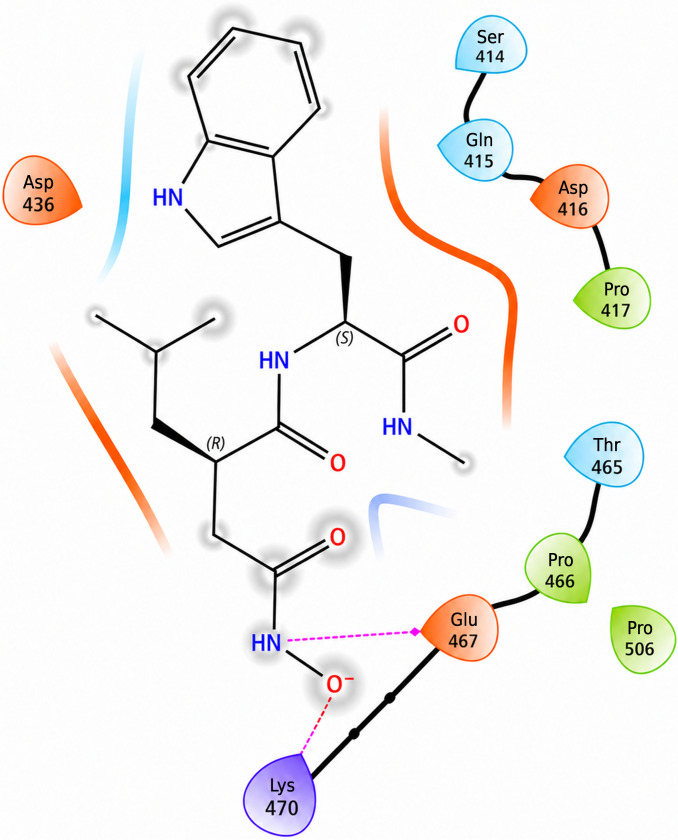
Two-dimensional interaction map of Ilomastat-2 binding to the MMP-2 active site. The map illustrates hydrogen bonding interactions with Asp416, Ser414, Gln415, Lys470, and Glu467, as well as hydrophobic contacts with Pro417 and Pro506, highlighting the key molecular interactions that contribute to Ilomastat-2’s stabilization and orientation within the catalytic pocket.

Electrostatic mapping revealed partial charge repulsion between Ilomastat-2 and nearby acidic residues (Fig. 8B), contributing to its reduced docking scores and decreased predicted stability. The interaction map (Fig. 9) indicates fewer stabilizing contacts overall, consistent with the less favorable Δ*G*_bind_ value.

**Fig. 10.**
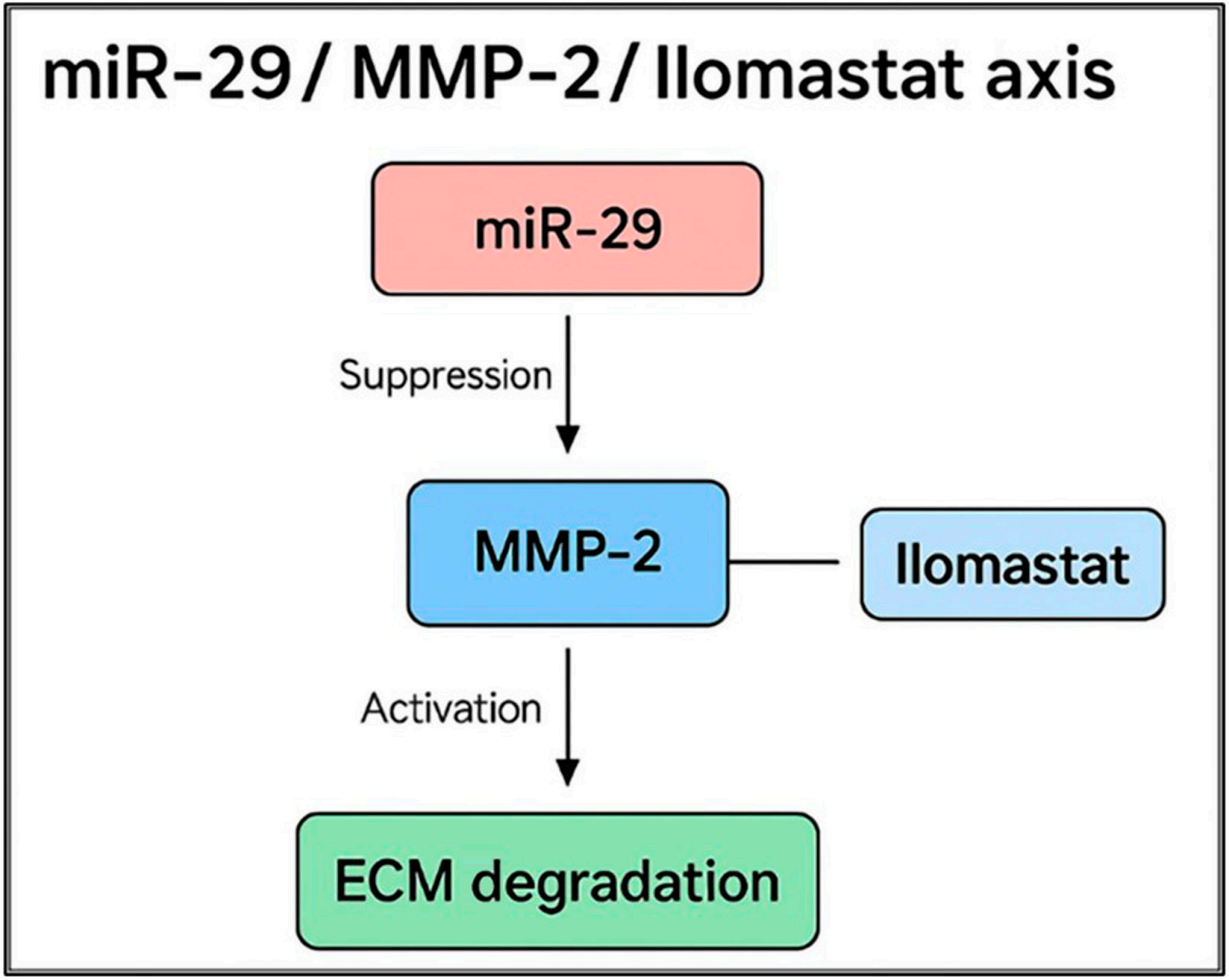
Schematic representation of the proposed miR-29/MMP-2/Ilomastat regulatory axis. Loss of miR-29 reduces the posttranscriptional repression of MMP-2, resulting in increased MMP-2 activity and enhanced ECM degradation. Ilomastat, particularly in its neutral tautomeric form, is predicted to bind within the catalytic Zn-containing site of MMP-2 and thereby provides a hypothesis-generating pharmacologic strategy for attenuating MMP-2 activity downstream of reduced miR-29-mediated repression, without implying structural or functional mimicry of miR-29 itself.

Together, these findings demonstrate that neutral Ilomastat binds more deeply, forms more extensive stabilizing interactions, and achieves superior energetic stability, supporting its consideration as a more energetically favorable candidate for MMP-2 inhibition within the proposed miR-29/MMP-2 axis (Fig. 10).

## Discussion

In this study, we comprehensively analyzed 29 paired South African ESCC samples, which enabled pathway-level miRNA enrichment analysis of the DEGs. This approach identified 9 candidate miRNAs, with the miR-29 family (including a/b/c-3p) emerging as the most significant (g:Profiler gSCS, *P* value ≤ 0.05). Across these samples, the up-regulation of MMP-2 (log₂FC = +1.42, *P* value = 0.037) was notably consistent with the loss of miR-29 repression, substantiating the previously hypothesized links between miRNA regulatory changes and downstream protease activity. *In silico* molecular docking of Ilomastat (GM6001) into the human MMP-2 catalytic pocket revealed pronounced binding preferences based on tautomeric state-neutral Δ*G*_bind_ ≈ –30.0 kcal·mol^−1^ versus anionic –19.9 kcal·mol^−1^ underscored by canonical zinc chelation and extensive multiresidue hydrogen bonding within the active site. Taken together, these findings provide integrative, hypothesis-generating evidence spanning transcriptomic miRNA enrichment and atomic-level effector inhibition, connecting miR-29 pathway disturbances to MMP-2 activity in ESCC. Notably, the transcriptome-to-structure methodology outlined here represents one of the first ESCC studies in an African cohort that suggests the relevance of miRNA-guided MMP-2 inhibition.

Our study advances a novel integrative perspective by explicitly bridging miRNA alterations with the structural inhibition of its protein effector target. The functional coupling between miR-29 down-regulation and enhanced MMP-2 activity is central to the pathobiology of ESCC, particularly because MMP-2 has a well-described role as a facilitator of tumor invasion and early metastatic dissemination. Through computational docking, we observed that Ilomastat can adopt a favorable pose within the catalytic pocket of MMP-2, including the zinc-binding region. These findings do not demonstrate direct mimicry or functional equivalence to miR-29, but they do provide a hypothesis-generating structural rationale for considering pharmacologic MMP-2 inhibition in the context of reduced miR-29-associated repression. Our data therefore extend the literature by providing indirect, integrative support derived from mRNA-level enrichment and computational docking for further investigation of this regulatory axis.

The integration of *in silico* docking with transcriptome-derived miRNA enrichment provides a structural framework for interpreting the potential relevance of the miR-29/MMP-2 axis in ESCC. Under physiological conditions, the miR-29 family is expected to attenuate MMP-2 expression via posttranscriptional regulation. In our study, docking suggested that Ilomastat may bind favorably within the MMP-2 catalytic site through zinc coordination and a stabilizing interaction network. However, these observations remain hypothesis-generating and do not by themselves establish biological activity, enzymatic inhibition, or functional compensation. The key findings of this study are as follows: first, the combination of transcriptomic enrichment and docking supports a plausible link between reduced miR-29-associated repression and MMP-2 overactivity; second, tautomer-specific docking effects indicate that protonation state may influence predicted inhibitor stability and binding energy; and third, these findings provide a rationale for future validation studies, including molecular dynamics simulations and direct enzymatic or cell-based assays, to evaluate MMP-2 inhibition in ESCC-relevant systems.

By combining transcriptomic enrichment and atomic-level structural modeling, our study revealed how miRNA malfunction, often a challenge for direct restoration in patients, may be complemented by rational small-molecule intervention targeting the same effector enzyme. Thus, the integration demonstrated in this study bridges the gap between genomic deregulation and targeted drug-level inhibition. These data do not demonstrate that Ilomastat phenocopies the miRNA-based inhibitory mechanism, but they do support its consideration as a structural lead for future studies of MMP-2-directed therapeutic design in ESCC.

### Enriched miRNAs

Bioinformatic analysis of 58 samples of paired tumor and matched adjacent normal tissues substantiated the relevance of several pathways, including miRNAs, KEGG signals, CORUM complexes, and GO:CC/MF/BP categories, in the regulation of ESCC-related DEGs. This analysis highlighted multiple miRNAs previously implicated in the molecular characteristics and clinical outcomes of ESCC. Dysregulation of these miRNAs is well-established to be associated with cancer development and progression, acting as either tumor suppressors or oncoMIRs in a context-dependent manner.

Several miRNAs were identified in this cohort through miRNA enrichment from mRNA-level differential expression using pathway analysis tools. These miRNAs have been implicated in ESCC and other malignancies. For instance, miR-335-5p displays variable expression in ESCC, gastric cancer (GC), hepatocellular carcinoma (HCC), colorectal cancer (CRC), and pancreatic carcinoma, and regulates invasion, migration, apoptosis, and proliferation, as well as modifies tumor cell radio- and chemosensitivity. In patients with GC, plasma hsa-miR-335-5p serves as a prognostic biomarker, with low levels correlating with poor outcomes [33]. In ESCC, miR-335 expression was significantly lower in malignant tissues (*P* value < 0.05) and inversely correlated with grade, lymph node status, and clinical stage. Multivariate models have identified miR-335 as a predictor of overall survival in ESCC patients [34]. In our local cohort, miR-335-5p was among the most enriched miRNAs (*P* value = 0.0), confirming its involvement in ESCC progression.

Our data identified miR-29 family members as top regulators of up-regulated DEGs [8]. The diagnostic and therapeutic targeting of these miRNAs could prove to be highly effective for ESCC management [35] in cancer therapy Previous studies have highlighted the critical nature of the reduced expression of miR-29b in ESCC. For example, miR-29b-3p was markedly lower in ESCC tissues than in adjacent normal tissues (*P* value < 0.05), and its overexpression suppressed MMP-2 protein levels and tumor growth in experimental settings [36]. Consistent with these reports, our pathway enrichment highlighted miR-29a/b/c-3p as very significant miRNAs (*P* value ≤ 0.05) in ESCC, with computational docking directly connecting this family to MMP-2, a validated target and effector in ESCC pathogenesis. Previous *in silico* prediction using miRTarBase also flagged miR-29a/b/c-3p and miR-26b-5p to be among the top regulators of up-regulated DEGs in esophageal cancer, reinforcing their biological relevance [8]. The miR-26 family directly influences the oncogenic MYC pathway directly targeting c-MYC and MYC-binding proteins (MYCBP), with miR-26a/b down-regulation shown to activate cell proliferation in ESCC tissues [35]. Restoration of their expression inhibits cancer growth, suggesting their clinical utility as tumor suppressors. These trends were confirmed in our dataset (miR-26b-5p enrichment, *P* value = 1e−04), highlighting the tumor-suppressive functions of this family.

miR-124-3p, another tumor suppressor, exerts regulatory activity across multiple cancers [37], whereas miR-122-5p acts as a context-dependent tumor suppressor [38–40]. In ESCC, promoter hypermethylation and down-regulation of MIR124-1 and MIR124-3 predicted a poor prognosis [41], while miR-122-5p specifically modulates ESCC cell proliferation and invasion via the miR-122/KIF22 axis [39]. In our enrichment data, the observed enrichment of both miR-124-3p (*P* value = 0.0223) and hsa-miR-122-5p (*P* value = 0.0378) was consistent with those of previous functional analyses.

Collectively, these findings reaffirm the contribution of tumor-suppressive miRNAs to the biology of ESCC. Early detection and combination of miRNA biomarker panels, including miR-29, miR-335, miR-26, miR-124, and miR-122, may improve diagnostic sensitivity [7]. Restoration of down-regulated miRNAs is a promising therapeutic avenue, with clinical studies demonstrating that elevating these repressed miRNAs reduces tumor progression and metastatic spread and improves ESCC prognosis [42].

### Computational docking and interpretation of binding plausibility

Computational docking of Ilomastat (GM6001), a broad-spectrum inhibitor of MMPs, provided structural insights into its predicted interactions with MMP-2 and the influence of protonation state on binding plausibility. MMP-2, a zinc-dependent endopeptidase, critically drives ECM degradation and contributes to tumor invasion and metastasis in ESCC [43,44]. Within the catalytic pocket, Zn^2+^ is coordinated by His403, His407, His413, and Glu404, forming a key locus for inhibitor interactions [44,45].

Energetic and conformational analyses indicated that the neutral tautomer (Ilomastat-1) adopted the more favorable binding pose, with stronger predicted affinity than the anionic tautomer (Ilomastat-2) (Glide SP –4.244 kcal·mol^−1^; XP –4.237 kcal·mol^−1^; MM-GBSA Δ*G*_bind_ –30.02 kcal·mol^−1^; Emodel –48.629 versus XP –2.804 kcal·mol^−1^; Δ*G*_bind_ –19.85 kcal·mol^−1^). These findings suggest that protonation state may influence predicted inhibitor stability and binding, with bidentate zinc coordination and hydrogen-bond stabilization contributing to the more favorable docking profile of the neutral tautomer [46,47].

Electrostatic potential mapping further supported these observations, showing a positively polarized cavity around Zn^2+^ that was more compatible with the modeled orientation of Ilomastat-1 than with the anionic tautomer [48–50]. Overall, the more favorable Δ*G*_bind_ predicted for Ilomastat-1 was consistent with its stronger modeled stabilization within the catalytic pocket, supporting the plausibility of the predicted binding mode. Comparative docking analyses also consistently favored Ilomastat-1 over the anionic tautomer, in keeping with its more favorable predicted interaction pattern within the MMP-2 active site.

Given MMP-2’s pathogenic contribution to ECM degradation and cancer progression, these findings support the biological plausibility of Ilomastat as a direct MMP-2 inhibitor candidate for further evaluation. This interpretation is consistent with prior non-ESCC experimental studies reporting Ilomastat-associated inhibition of MMP-2 or gelatinase activity, although such evidence does not substitute for validation in the present ESCC context [25,51]. Although docking and MM-GBSA rescoring provided consistent energetic support for the protonation-dependent stability of Ilomastat within the MMP-2 active site, no explicit molecular dynamics simulations were performed. Consequently, the present study does not capture time-dependent interactions such as water-mediated stabilization, Zn^2+^ coordination fluctuations, or side-chain rearrangements that may occur under physiological conditions. Short molecular dynamics simulations would therefore be valuable in future studies to evaluate the persistence of the predicted Ilomastat–MMP-2 interactions and refine the mechanistic interpretation

### LINC00392 and FABP4 overexpression

FABP4’s pronounced overexpression in tumor tissues highlights its pivotal role in orchestrating lipid metabolic reprogramming within the tumor microenvironment, a process critical for sustaining cancer cell proliferation and survival [52–54]. FABP4 supports the enhanced energetic and biosynthetic demands of aggressive tumor cells by facilitating fatty acid transport and storage [53,55]. This metabolic shift not only promotes tumor growth but also influences the surrounding stromal and immune cells, potentially creating an immunosuppressive milieu that favors tumor progression and metastasis [52,53,56]. Modulating the immunosuppressive tumor microenvironment is critical for the successful deployment of treatment techniques, such as immunotherapy. Zhang *et al.* discovered 121 MEM-related genes in the ESCC microenvironment that are linked to metabolic reprogramming that results in immunosuppression. They discovered that macrophages, T cells, epithelial cells, and B cells were high-scoring cells that expressed MEM-related genes, and consequently, they successfully developed a 4-gene prognostic model. This model revealed APOE and MAP1LC3A as possible target MEM-related genes for the development of anti-ESCC therapies [10]. Previous research has demonstrated that FABP4 expression is not statistically significant in esophageal cancer when comparing normal and malignant tissue [52], while Lin *et al.* [57] demonstrated using Western blot an increased expression of FABP4 in gastric adenocarcinoma cells compared to their adjacent normal tissues. The specific up-regulation of FABP4 in our ESCC patients may reflect distinct metabolic vulnerabilities or adaptations shaped by genetic, environmental, or lifestyle factors unique to this population. However, this overexpression needs to be further evaluated through direct measurement methodologies in our ESCC population group.

The association of FABP4 with increased invasiveness and metastatic potential suggests that it could serve as both a biomarker and a therapeutic target [52,54,55]. Targeting FABP4-mediated pathways may disrupt the metabolic crosstalk between tumor cells and their microenvironment, thereby impairing tumor aggressiveness and improving treatment outcomes [52,53]. Integrating FABP4 expression data with other DEGs could facilitate the development of precision medicine strategies tailored to the metabolic and immunological landscape of ESCC tumors. Such approaches might include inhibitors of lipid metabolism or combinatorial therapies that simultaneously address metabolic reprogramming and immune evasion mechanisms, offering new avenues for combating this malignancy [52,53,56].

There is growing evidence that long noncoding RNAs (lncRNAs) play a pivotal role in esophageal cancer. In their analysis of 551 gene expression profiles of normal, ESCC, and EAC tissues or cell lines, Li *et al.* found 1,159 differentially expressed lncRNAs that were crucial for the development of both cancer subtypes [58]. LINC00392 is a long intergenic nonprotein-coding RNA 392 gene found on chromosome 13 in humans. It was found to be highly expressed (fold change > 5) in ESCC using RNA-seq [59,60]. It has also been identified as an independent prognostic factor in gastric cancer [61] and is differentially expressed in lung adenocarcinoma [62]. Integration of the HPV genome into the host genome is a key event in cervical cancer carcinogenesis. Zhou *et al.* used long-read sequencing nanopore technology to undertake comprehensive characterization of large-range virus–human integration events in 16 HPV16-positive primary cervical tumors. They identified a recurrent hotspot integration region on chromosome 13 between the KLF5 and LINC00392 genes, which disrupted these genes [63]. Approximately 72.4% of our patients tested positive for HPV, with HPV16 being the most common strain (58.6%). The substantial overexpression of LINC00392 in our study could also be partly attributable to the similar disruption produced by HPV viral integration; however, further validation experiments are needed.

## Limitations

Although our strategy was successful in identifying potential miRNA-associated regulatory pathways, the RNA-seq methodology used to prepare the sample libraries was primarily optimized for large RNAs. Consequently, miR-29 dysregulation in this cohort was inferred from PEA rather than measured directly at the miRNA level. Future studies using small RNA-seq are therefore needed for comprehensive profiling and direct validation of the implicated miRNAs.

An additional limitation relates to the docking analysis. The docking results presented here predict plausible binding poses and relative energetic favorability for Ilomastat within the MMP-2 catalytic site, but they do not by themselves establish biological activity, enzymatic inhibition, or functional compensation for reduced miR-29 activity. No molecular dynamics simulations or direct MMP-2 enzymatic inhibition assays were performed in the present study; these should be considered important next steps for evaluating the stability and functional relevance of the predicted docking poses.

While FABP4 and LINC00392 were highly expressed in tumor tissues, our study was conducted on bulk tumor samples compared with adjacent normal mucosa. Sample dissociation, cell labeling, and bioinformatic deconvolution were not performed prior to analysis; therefore, these substantial overexpression signals should be interpreted in that context. In particular, FABP4 overexpression may partly reflect tumor-infiltrating macrophages, adipose contamination, or stromal and vascular components.

As in many RNA-seq studies, our sample size was relatively small, comprising 29 paired samples (58 total samples). RNA-seq experiments commonly face sample-size constraints due to limitations in funding, personnel, and infrastructure. Small cohort size can reduce replicability and recall in differential expression and pathway enrichment analyses, even though this does not necessarily imply a high false-positive rate or poor enrichment precision [64].

Another major limitation of our study is the lack of orthogonal validation, such as RT-qPCR, immunohistochemistry, or Western blotting, to support the next-generation sequencing and docking findings. These limitations were primarily related to limited available resources and should be addressed in future validation studies. In addition, we did not perform *in silico* validation using external RNA-seq cohorts, such as TCGA ESCA, which should be explored in future studies.

## Conclusion

This study presents an integrative framework linking patient-derived mRNA transcriptomic data to structure-level modeling of MMP-2 targeting in ESCC. Pathway enrichment among paired South African ESCC samples highlighted the miR-29 family as probable regulators of key DEGs, and reduced miR-29-associated repression was mechanistically consistent with increased MMP-2 activity. Computational docking analyses suggested that Ilomastat can plausibly bind the catalytic site of MMP-2 in a manner consistent with downstream pharmacologic inhibition. These observations are hypothesis-generating and do not establish direct mimicry of miR-29 or functional compensation for reduced miR-29 activity. Rather, they provide a structural rationale for future studies integrating molecular dynamics simulations, direct MMP-2 enzymatic assays, and cell-based validation to test the biological relevance of this proposed axis in ESCC.

## Future Research

We regard our findings as laying the groundwork for additional directed investigations that will target these miRNAs through comprehensive, dedicated sequencing and/or PCR-based approaches and examine the clinicopathological variables associated with their expression**.**

## Ethical Approval

Ethical approval was obtained from the University of Pretoria Research Ethics Committee (Study No. 296/2021) and Gauteng Health Department (NHRD Ref No. GP_202107_062). The study was conducted in accordance with local legislation and institutional requirements. All participants provided written informed consent to participate in the study.

## Data Availability

Raw data were generated by the National Institute for Communicable Diseases. The derived data supporting the findings of this study are available from the corresponding authors (S.Z.M.) and Z.D. on request.

## References

[1] Zarrilli G, Galuppini F, Angerilli V, Munari G, Sabbadin M, Lazzarin V, Nicolè L, Biancotti R, Fassan M. miRNAs involved in esophageal carcinogenesis and miRNA-related therapeutic perspectives in esophageal carcinoma. Int J Mol Sci. 2021;22(7): Article 3640. 33807389 10.3390/ijms22073640 PMC8037581

[2] Miyoshi J, Zhu Z, Luo A, Toden S, Zhou X, Izumi D, Kanda M, Takayama T, Parker IM, Wang M, et al. A microRNA-based liquid biopsy signature for the early detection of esophageal squamous cell carcinoma: A retrospective, prospective and multicenter study. Mol Cancer. 2022;21(1):44. 35148754 10.1186/s12943-022-01507-x PMC8832722

[3] Bhaskaran M, Mohan M. MicroRNAs: History, biogenesis, and their evolving role in animal development and disease. Vet Pathol. 2014;51(4):759–774. 24045890 10.1177/0300985813502820 PMC4013251

[4] Shang R, Lee S, Senavirathne G, Lai EC. microRNAs in action: Biogenesis, function and regulation. Nat Rev Genet. 2023;24(12):816–833. 37380761 10.1038/s41576-023-00611-y PMC11087887

[5] Svoronos AA, Engelman DM, Slack FJ. OncomiR or tumor suppressor? The duplicity of microRNAs in cancer. Cancer Res. 2016;76(13):3666–3670. 27325641 10.1158/0008-5472.CAN-16-0359 PMC4930690

[6] Zheng Q, Hou W. Regulation of angiogenesis by microRNAs in cancer. Mol Med Rep. 2021;24(2): Article 583. 34132365 10.3892/mmr.2021.12222 PMC8223106

[7] He Z, Ji Y, Yuan Y, Liang T, Liu C, Jiao Y, Chen Y, Yang Y, Han L, Hu Y, et al. Uncovering the role of microRNAs in esophageal cancer: From pathogenesis to clinical applications. Front Pharmacol. 2025;16: Article 1532558. 39944625 10.3389/fphar.2025.1532558 PMC11814179

[8] Mokhlesi A, Sharifi Z, Berimipour A, Taleahmad S, Talkhabi M. Identification of hub genes and microRNAs with prognostic values in esophageal cancer by integrated analysis. Non-coding RNA Res. 2023;8(3):459–470. 10.1016/j.ncrna.2023.05.009 PMC10319852 37416747

[9] Barchi A, Massimino L, Mandarino FV, Vespa E, Sinagra E, Almolla O, Passaretti S, Fasulo E, Parigi TL, Cagliani S, et al. Microbiota profiling in esophageal diseases: Novel insights into molecular staining and clinical outcomes. Comput Struct Biotechnol J. 2024;23:626–637. 38274997 10.1016/j.csbj.2023.12.026 PMC10808859

[10] Zhang Z, Jin G, Zhao J, Deng S, Chen F, Wuyun G, Zhao L, Li Q. Mitochondrial energy metabolism correlates with an immunosuppressive tumor microenvironment and poor prognosis in esophageal squamous cell carcinoma. Comput Struct Biotechnol J. 2023;21:4118–4133. 37664173 10.1016/j.csbj.2023.08.022 PMC10474161

[11] Shaham L, Binder V, Gefen N, Borkhardt A, Izraeli S. MiR-125 in normal and malignant hematopoiesis. Leukemia. 2012;26(9):2011–2018. 22456625 10.1038/leu.2012.90

[12] Szabados T, Makkos A, Ágg B, Benczik B, Brenner GG, Szabó M, Váradi B, Vörös I, Gömöri K, Varga ZV, et al. Pharmacokinetics and cardioprotective efficacy of intravenous miR-125b* microRNA mimic in a mouse model of acute myocardial infarction. Br J Pharmacol. 2025;182(2):432–450. 39472767 10.1111/bph.17345

[13] Mei LL, Wang WJ, Qiu YT, Xie XF, Bai J, Shi ZZ. miR-125b-5p functions as a tumor suppressor gene partially by regulating HMGA2 in esophageal squamous cell carcinoma. PLOS ONE. 2017;12(10): Article e0185636. 28968424 10.1371/journal.pone.0185636 PMC5624607

[14] Ossandon FJ, Villarroel C, Aguayo F, Santibanez E, Oue N, Yasui W, Corvalan AH. In silico analysis of gastric carcinoma serial analysis of gene expression libraries reveals different profiles associated with ethnicity. Mol Cancer. 2008;7:22. 18302799 10.1186/1476-4598-7-22 PMC2323622

[15] Smyth EC, Lagergren J, Fitzgerald RC, Lordick F, Shah MA, Lagergren P, Cunningham D. Oesophageal cancer. Nat Rev Dis Primers. 2017;3(1):17048. 28748917 10.1038/nrdp.2017.48 PMC6168059

[16] Reddy RA, Varshini MS, Kumar RS. Matrix metalloproteinase-2 (MMP-2): As an essential factor in cancer progression. Recent Pat Anticancer Drug Discov. 2025;20(1):26–44. 37861020 10.2174/0115748928251754230922095544

[17] Yu D, Lai P, Yan T, Fang K, Chen L, Zhang S. Quantifying the matrix metalloproteinase 2 (MMP2) spatially in tissues by probe via MALDI imaging mass spectrometry. Front Chem. 2021;9: Article 786283. 34976953 10.3389/fchem.2021.786283 PMC8715900

[18] Asombang AW, Chishinga N, Nkhoma A, Chipaila J, Nsokolo B, Manda-Mapalo M, Montiero JFG, Banda L, Dua KS. Systematic review and meta-analysis of esophageal cancer in Africa: Epidemiology, risk factors, management and outcomes. World J Gastroenterol. 2019;25(31):4512–4533. 31496629 10.3748/wjg.v25.i31.4512 PMC6710188

[19] Bandidwattanawong C. Multi-disciplinary management of esophageal carcinoma: Current practices and future directions. Crit Rev Oncol Hematol. 2024;197: Article 104315. 38462149 10.1016/j.critrevonc.2024.104315

[20] Obermannová R, Alsina M, Cervantes A, Leong T, Lordick F, Nilsson M, van Grieken N, Vogel A, Smyth EC, ESMO Guidelines Committee. Electronic address: clinicalguidelines@esmo.org. Oesophageal cancer: ESMO clinical practice guideline for diagnosis, treatment and follow-up. Ann Oncol. 2022;33(10):992–1004. 35914638 10.1016/j.annonc.2022.07.003

[21] Li Y, Ma J, Guo Q, Duan F, Tang F’, Zheng P, Zhao Z, Lu G. Overexpression of MMP-2 and MMP-9 in esophageal squamous cell carcinoma. Dis Esophagus. 2009;22(8):664–667. 19191857 10.1111/j.1442-2050.2008.00928.x

[22] Celentano A, Yap T, Paolini R, Yiannis C, Mirams M, Koo K, McCullough M, Cirillo N. Inhibition of matrix metalloproteinase-2 modulates malignant behaviour of oral squamous cell carcinoma cells. J Oral Pathol Med. 2021;50(3):323–332. 31925966 10.1111/jop.12992

[23] Kim JH, Jeon S, Shin BA. MicroRNA-29 family suppresses the invasion of HT1080 human fibrosarcoma cells by regulating matrix metalloproteinase 2 expression. Chonnam Med J. 2017;53(2):161–167. 28584796 10.4068/cmj.2017.53.2.161 PMC5457952

[24] Fang J-H, Zhou H-C, Zeng C, Yang J, Liu Y, Huang X, Zhang JP, Guan XY, Zhuang SM. MicroRNA-29b suppresses tumor angiogenesis, invasion, and metastasis by regulating matrix metalloproteinase 2 expression. Hepatology. 2011;54(5):1729–1740. 21793034 10.1002/hep.24577

[25] Bencsik P, Pálóczi J, Kocsis GF, Pipis J, Belecz I, Varga ZV, Csonka C, Görbe A, Csont T, Ferdinandy P. Moderate inhibition of myocardial matrix metalloproteinase-2 by ilomastat is cardioprotective. Pharmacol Res. 2014;80:36–42. 24380772 10.1016/j.phrs.2013.12.007

[26] Ewels PA, Peltzer A, Fillinger S, Patel H, Alneberg J, Wilm A, Garcia MU, di Tommaso P, Nahnsen S. The nf-core framework for community-curated bioinformatics pipelines. Nat Biotechnol. 2020;38(3):276–278. 32055031 10.1038/s41587-020-0439-x

[27] Grüning B, Dale R, Sjödin A, Chapman BA, Rowe J, Tomkins-Tinch CH, Valieris R, Koster J, Team B. Bioconda: Sustainable and comprehensive software distribution for the life sciences. Nat Methods. 2018;15(7):475–476. 29967506 10.1038/s41592-018-0046-7 PMC11070151

[28] da Veiga F, Grüning BA, Alves Aflitos S, Röst HL, Uszkoreit J, Barsnes H, Vaudel M, Moreno P, Gatto L, Weber J, et al. BioContainers: An open-source and community-driven framework for software standardization. Bioinformatics. 2017;33(16):2580–2582. 28379341 10.1093/bioinformatics/btx192 PMC5870671

[29] Di Tommaso P, Chatzou M, Floden EW, Barja PP, Palumbo E, Notredame C. Nextflow enables reproducible computational workflows. Nat Biotechnol. 2017;35(4):316–319. 28398311 10.1038/nbt.3820

[30] Kolberg L, Raudvere U, Kuzmin I, Vilo J, Peterson H. gprofiler2—An R package for gene list functional enrichment analysis and namespace conversion toolset g:Profiler. F1000Res. 2020;9: Article ELIXIR-709. 10.12688/f1000research.24956.1 PMC7859841 33564394

[31] Chicco D, Agapito G. Nine quick tips for pathway enrichment analysis. PLOS Comput Biol. 2022;18(8): Article e1010348. 35951505 10.1371/journal.pcbi.1010348 PMC9371296

[32] Reimand J, Kull M, Peterson H, Hansen J, Vilo J. G:Profiler—A web-based toolset for functional profiling of gene lists from large-scale experiments. Nucleic Acids Res. 2007;35(Web Server issue):W193–W200. 17478515 10.1093/nar/gkm226 PMC1933153

[33] Sandoval-Bórquez A, Polakovicova I, Carrasco-Véliz N, Lobos-González L, Riquelme I, Carrasco-Avino G, Bizama C, Norero E, Owen GI, Roa JC, et al. MicroRNA-335-5p is a potential suppressor of metastasis and invasion in gastric cancer. Clin Epigenetics. 2017;9(1):114. 29075357 10.1186/s13148-017-0413-8 PMC5645854

[34] Zhang BJ, Gong HY, Zheng F, Liu DJ, Liu HX. Up-regulation of miR-335 predicts a favorable prognosis in esophageal squamous cell carcinoma. Int J Clin Exp Pathol. 2014;7(9):6213–6218. 25337272 PMC4203243

[35] Li J, Liang Y, Lv H, Meng H, Xiong G, Guan X, Chen X, Bai Y, Wang K. miR-26a and miR-26b inhibit esophageal squamous cancer cell proliferation through suppression of c-MYC pathway. Gene. 2017;625:1–9. 28476684 10.1016/j.gene.2017.05.001

[36] Qi Y, Li X, Zhao S. miR-29b inhibits the progression of esophageal squamous cell carcinoma by targeting MMP-2. Neoplasma. 2015;62(3):384–390. 25866219 10.4149/neo_2015_046

[37] Long HD, Ma YS, Yang HQ, Xue SB, Liu JB, Yu F, Lv ZW, Li JY, Xie RT, Chang ZY, et al. Reduced hsa-miR-124-3p levels are associated with the poor survival of patients with hepatocellular carcinoma. Mol Biol Rep. 2018;45(6):2615–2623. 30341691 10.1007/s11033-018-4431-1

[38] Faramin Lashkarian M, Hashemipour N, Niaraki N, Soghala S, Moradi A, Sarhangi S, Hatami M, Aghaei-Zarch F, Khosravifar M, Mohammadzadeh A, et al. MicroRNA-122 in human cancers: From mechanistic to clinical perspectives. Cancer Cell Int. 2023;23(1):29. 36803831 10.1186/s12935-023-02868-z PMC9940444

[39] Wang J, Yu PY, Yu JP, Luo JD, Sun ZQ, Sun F, Kong Z, Wang JL. KIF22 promotes progress of esophageal squamous cell carcinoma cells and is negatively regulated by miR-122. Am J Transl Res. 2021;13(5):4152–4166. 34150005 PMC8205736

[40] Dai C, Zhang Y, Xu Z, Jin M. MicroRNA-122-5p inhibits cell proliferation, migration and invasion by targeting CCNG1 in pancreatic ductal adenocarcinoma. Cancer Cell Int. 2020;20(1):98. 32256207 10.1186/s12935-020-01185-z PMC7106816

[41] Tian Z, Li Z, Zhu Y, Meng L, Liu F-f, Sang M, Wang G. Hypermethylation-mediated inactivation of miR-124 predicts poor prognosis and promotes tumor growth at least partially through targeting EZH2/H3K27me3 in ESCC. Clin Exp Metastasis. 2019;36:381–391. 31197517 10.1007/s10585-019-09974-1

[42] Liu W-J, Zhao Y, Chen X, Miao M-L, Zhang R-Q. Epigenetic modifications in esophageal cancer: An evolving biomarker. Front Genet. 2023;13: Article 1087479. 36704345 10.3389/fgene.2022.1087479 PMC9871503

[43] Bergers G, Brekken R, McMahon G, Vu TH, Itoh T, Tamaki K, Tanzawa K, Thorpe P, Itohara S, Werb Z, et al. Matrix metalloproteinase-9 triggers the angiogenic switch during carcinogenesis. Nat Cell Biol. 2000;2(10):737–744. 11025665 10.1038/35036374 PMC2852586

[44] Kleifeld O, Kotra LP, Gervasi DC, Brown S, Bernardo MM, Fridman R, Mobashery S, Sagi I. X-ray absorption studies of human matrix metalloproteinase-2 (MMP-2) bound to a highly selective mechanism-based inhibitor. Comparison with the latent and active forms of the enzyme. J Biol Chem. 2001;276(20):17125–17131. 11278946 10.1074/jbc.M011604200

[45] Kleifeld O, van den Steen PE, Frenkel A, Cheng F, Jiang HL, Opdenakker G, Sagi I. Structural characterization of the catalytic active site in the latent and active natural gelatinase B from human neutrophils. J Biol Chem. 2000;275(44):34335–34343. 10938090 10.1074/jbc.M005714200

[46] Tao P, Fisher JF, Shi Q, Mobashery S, Schlegel HB. Matrix metalloproteinase 2 (MMP2) inhibition: DFT and QM/MM studies of the deprotonation-initialized ring-opening reaction of the sulfoxide analogue of SB-3CT. J Phys Chem B. 2010;114(2):1030–1037. 20039633 10.1021/jp909327y PMC2821710

[47] Heikkilä P, Suojanen J, Pirilä E, Väänänen A, Koivunen E, Sorsa T, Salo T. Human tongue carcinoma growth is inhibited by selective antigelatinolytic peptides. Int J Cancer. 2006;118(9):2202–2209. 16331606 10.1002/ijc.21540

[48] Dunten P, Kammlott U, Crowther R, Levin W, Foley LH, Wang P, Palermo R. X-ray structure of a novel matrix metalloproteinase inhibitor complexed to stromelysin. Protein Sci. 2001;10(5):923–926. 11316871 10.1110/ps.48401 PMC2374200

[49] Hernandez-Barrantes S, Toth M, Bernardo MM, Yurkova M, Gervasi DC, Raz Y, Sang QXA, Fridman R. Binding of active (57 kDa) membrane type 1-matrix metalloproteinase (MT1-MMP) to tissue inhibitor of metalloproteinase (TIMP)-2 regulates MT1-MMP processing and pro-MMP-2 activation. J Biol Chem. 2000;275(16):12080–12089. 10766841 10.1074/jbc.275.16.12080

[50] Yang M, Huang H, Li J, Huang W, Wang H. Connective tissue growth factor increases matrix metalloproteinase-2 and suppresses tissue inhibitor of matrix metalloproteinase-2 production by cultured renal interstitial fibroblasts. Wound Repair Regen. 2007;15(6):817–824. 18028129 10.1111/j.1524-475X.2007.00284.x

[51] Bencsik P, Kupai K, Giricz Z, Görbe A, Pipis J, Murlasits Z, Kocsis GF, Varga-Orvos Z, Puskás LG, Csonka C, et al. Role of iNOS and peroxynitrite-matrix metalloproteinase-2 signaling in myocardial late preconditioning in rats. Am J Physiol Heart Circ Physiol. 2010;299(2):H512–H518. 20543091 10.1152/ajpheart.00052.2010

[52] Yang J, Liu X, Shao Y, Zhou H, Pang L, Zhu W. Diagnostic, prognostic, and immunological roles of FABP4 in pancancer: A bioinformatics analysis. Comput Math Methods Med. 2022;2022:3764914. 36532833 10.1155/2022/3764914 PMC9754845

[53] Yorek M, Jiang X, Liu S, Hao J, Yu J, Avellino A, Liu Z, Curry M, Keen H, Shao J, et al. FABP4-mediated lipid accumulation and lipolysis in tumor associated macrophages promote breast cancer metastasis. bioRxiv. 2024. https://doi.org/10.1101/2024.07.02.601733 10.7554/eLife.101221 PMC11548877 39513934

[54] Gharpure KM, Pradeep S, Sans M, Rupaimoole R, Ivan C, Wu SY, Bayraktar E, Nagaraja AS, Mangala LS, Zhang X, et al. FABP4 as a key determinant of metastatic potential of ovarian cancer. Nat Commun. 2018;9(1):2923. 30050129 10.1038/s41467-018-04987-y PMC6062524

[55] Wu D, Xiang L, Peng L, Gu H, Tang Y, Luo H, Liu H, Wang Y. Comprehensive analysis of the immune implication of FABP4 in colon adenocarcinoma. PLOS ONE. 2022;17(10): Article e0276430. 36264920 10.1371/journal.pone.0276430 PMC9584364

[56] Zhang D, Wang M, Liu G, Li X, Yu W, Hui Z, Ren X, Sun Q. Novel FABP4+C1q+ macrophages enhance antitumor immunity and associated with response to neoadjuvant pembrolizumab and chemotherapy in NSCLC via AMPK/JAK/STAT axis. Cell Death Dis. 2024;15(10):717. 39353883 10.1038/s41419-024-07074-x PMC11445384

[57] Lin R, Zhang H, Yuan Y, He Q, Zhou J, Li S, Sun Y, Li DY, Qiu HB, Wang W, et al. Fatty acid oxidation controls CD8+ tissue-resident memory T-cell survival in gastric adenocarcinoma. Cancer Immunol Res. 2020;8(4):479–492. 32075801 10.1158/2326-6066.CIR-19-0702

[58] Li X, Wang Y, Min Q, Zhang W, Teng H, Li C, Zhang K, Shi L, Wang B, Zhan Q. Comparative transcriptome characterization of esophageal squamous cell carcinoma and adenocarcinoma. Comput Struct Biotechnol J. 2023;21:3841–3853. 37564101 10.1016/j.csbj.2023.07.030 PMC10410469

[59] Pan Z, Mao W, Bao Y, Zhang M, Su X, Xu X. The long noncoding RNA CASC9 regulates migration and invasion in esophageal cancer. Cancer Med. 2016;5(9):2442–2447. 27431358 10.1002/cam4.770 PMC5055159

[60] Li C, Yao W, Zhao C, Yang G, Wei J, Qi Y, Huang R, Zhao Q, Hao C. Comprehensive analysis of lncRNAs related to the prognosis of esophageal cancer based on ceRNA network and cox regression model. Biomed Res Int. 2020;2020:3075729. 33381546 10.1155/2020/3075729 PMC7748909

[61] Guo J, Liu Y, Zhao P. Bioinformatic analysis identified potentially prognostic long noncoding RNAs and microRNAs for gastric cancer. Biomed Res Int. 2021;2021:6683136. 34926687 10.1155/2021/6683136 PMC8683174

[62] Hu J, Wang T, Chen Q. Competitive endogenous RNA network identifies four long non-coding RNA signature as a candidate prognostic biomarker for lung adenocarcinoma. Transl Cancer Res. 2019;8(4):1046–1064. 35116848 10.21037/tcr.2019.06.09 PMC8798056

[63] Zhou L, Qiu Q, Zhou Q, Li J, Yu M, Li K, Xu L, Ke X, Xu H, Lu B, et al. Long-read sequencing unveils high-resolution HPV integration and its oncogenic progression in cervical cancer. Nat Commun. 2022;13(1):2563. 35538075 10.1038/s41467-022-30190-1 PMC9091225

[64] Degen PM, Medo M. Replicability of bulk RNA-seq differential expression and enrichment analysis results for small cohort sssizes. PLOS Comput Biol. 2025;21(5): Article e1011630. 40324149 10.1371/journal.pcbi.1011630 PMC12077797

