# Supplementary material for: MicroRNA Enrichment and Docking-Based Evaluation of Ilomastat Targeting of MMP-2 in Esophageal Squamous Cell Carcinoma: Insights from a South African Cohort
Source: Comput Struct Biotechnol J. 2026 May 18;35(1):0097. doi: 10.34133/csbj.0097 (PMC13181172; doi:10.34133/csbj.0097)
Supplement: Supplementary 1 — Table S1 [file csbj.0097.f1.docx]

**Supplementary Data**

**Table S1: Total RNA quality control. Results of quality control measures assessing the concentrations and integrity of the extracted RNA.**

|  | **Nanodrop – BioDrop Flourometer** | | | **Qubit 4 spectrophotometry** | | |
| --- | --- | --- | --- | --- | --- | --- |
| **Sample ID** | **Concentration (ng/µl)** | **A260/A230** | **A260/A280** | **IQ Score** | **Small RNA** | **Large or Structured RNA** |
| **OES 67 Normal** | **370.4** | **1.7** | **2.0** | **9.1** | **9%** | **91%** |
| **OES 67 Tumour** | **735.2** | **2.0** | **2.0** | **8.3** | **17%** | **83%** |
| **OES 69 Normal** | **379.2** | **0.7** | **1.8** | **6.4** | **36%** | **64%** |
| **OES 69 Tumour** | **588.8** | **2.1** | **2.0** | **8.7** | **13%** | **87%** |
| **OES 70 Normal** | **210** | **1.8** | **2.0** | **8.6** | **14%** | **86%** |
| **OES 70 Tumour** | **248.8** | **1.9** | **2.0** | **8.8** | **12%** | **88%** |
| **OES 71 Normal** | **471.2** | **1.6** | **2.0** | **9.1** | **9%** | **91%** |
| **OES 71 Tumour** | **480.8** | **2.1** | **2.0** | **8.5** | **15%** | **85%** |
| **OES 72 Normal** | **445.6** | **1.7** | **2.0** | **8.9** | **11%** | **89%** |
| **OES 72 Tumour** | **515.2** | **1.6** | **2.0** | **8.6** | **14%** | **86%** |
| **OES 73 Normal** | **244.8** | **1.9** | **2.0** | **8.7** | **13%** | **87%** |
| **OES 73 Tumour** | **399.2** | **1.9** | **2.0** | **9.0** | **10%** | **90%** |
| **OES 74B Normal** | **328** | **1.8** | **2.0** | **9.4** | **6%** | **94%** |
| **OES74B Tumour** | **295.2** | **1.9** | **2.0** | **8.4** | **16%** | **84%** |
| **OES 78 Normal** | **322.4** | **1.9** | **2.0** | **9.2** | **8%** | **92%** |
| **OES 78 Tumour** | **687.2** | **2.1** | **2.0** | **7.9** | **21%** | **79%** |
| **OES 80A Normal** | **565.6** | **1.7** | **2.0** | **8.8** | **12%** | **88%** |
| **OES80A Tumour** | **917.6** | **2.0** | **2.0** | **9.1** | **9%** | **91%** |
| **OES 81 Normal** | **584** | **2.1** | **2.1** | **9.1** | **9%** | **91%** |
| **OES 81 Tumour** | **608** | **1.9** | **2.1** | **9.5** | **5%** | **95%** |
| **OES 82 Normal** | **824** | **2.0** | **2.1** | **10.0** | **0%** | **100%** |
| **OES 82 Tumour** | **428** | **2.1** | **2.1** | **9.7** | **3%** | **97%** |
| **OES 85 Normal** | **433** | **1.9** | **2.0** | **10** | **0%** | **100%** |
| **OES 85 Tumour** | **302** | **2.1** | **2.0** | **9.4** | **6%** | **94%** |
| **OES 86 Normal** | **1181.6** | **2.1** | **2.1** | **8.5** | **15%** | **85%** |
| **OES 86 Tumour** | **820** | **2.1** | **2.0** | **8.9** | **11%** | **89%** |
| **OES 87 Normal** | **580.8** | **1.7** | **2.0** | **8.4** | **16%** | **84%** |
| **OES 87 Tumour** | **677.6** | **2.0** | **2.0** | **8.2** | **18%** | **82%** |
| **OES 89 Normal** | **897.6** | **1.7** | **2.0** | **8.9** | **11%** | **89%** |
| **OES 89 Tumour** | **520.8** | **2.1** | **2.0** | **8.3** | **17%** | **83%** |
| **OES 90 Normal** | **1213.5** | **1.9** | **2.0** | **8.4** | **16%** | **84%** |
| **OES 90 Tumour** | **704** | **1.9** | **2.0** | **8.2** | **18%** | **82%** |
| **OES 91 Normal** | **1402.4** | **2.0** | **2.0** | **8.1** | **19%** | **81%** |
| **OES 91 Tumour** | **766.4** | **2.1** | **2.0** | **9.2** | **8%** | **92%** |
| **OES 92 Normal** | **570.4** | **2.1** | **2.0** | **8.9** | **11%** | **89%** |
| **OES 92 Tumour** | **672** | **2.0** | **1.9** | **8.5** | **15%** | **85%** |
| **OES 94 Normal** | **572** | **1.8** | **2.0** | **9.1** | **9%** | **91%** |
| **OES 94 Tumour** | **1432.8** | **2.1** | **2.0** | **7.8** | **22%** | **78%** |
| **OES 95 Normal** | **968** | **2.0** | **2.0** | **9.0** | **10%** | **90%** |
| **OES 95 Tumour** | **871.2** | **2.1** | **2.0** | **8.8** | **12%** | **88%** |
| **OES 96 Normal** | **487.2** | **1.8** | **2.0** | **9.0** | **10%** | **90%** |
| **OES 96 Tumour** | **820** | **1.9** | **2.0** | **9.1** | **9%** | **91%** |
| **OES 97 Normal** | **988** | **2.1** | **2.0** | **8.9** | **11%** | **89%** |
| **OES 97 Tumour** | **512.8** | **2.0** | **1.9** | **5.8** | **42%** | **58%** |
| **OES 98 Normal** | **418.4** | **1.9** | **2.0** | **9.3** | **7%** | **93%** |
| **OES 98 Tumour** | **898.4** | **2.0** | **2.0** | **8.9** | **11%** | **89%** |
| **OES 99 Normal** | **1196** | **1.9** | **2.0** | **7.9** | **21%** | **79%** |
| **OES 99 Tumour** | **1188** | **1.8** | **2.0** | **8.6** | **14%** | **86%** |
| **OES 100 Normal** | **699.2** | **2.1** | **2.0** | **8.1** | **19%** | **81%** |
| **OES100 Tumour** | **1065.6** | **2.2** | **2.0** | **8.8** | **12%** | **88%** |
| **OES 101 Normal** | **220.8** | **18** | **2.0** | **9.6** | **4%** | **96%** |
| **OES 101 Tumour** | **336.8** | **1.8** | **2.0** | **8.4** | **16%** | **84%** |
| **OES 102 Normal** | **346.4** | **2.0** | **2.1** | **9.7** | **3%** | **97%** |
| **OES 102 Tumour** | **374.4** | **1.8** | **2.1** | **9.6** | **4%** | **96%** |
| **OES 103 Normal** | **523.2** | **1.7** | **2.0** | **9.3** | **7%** | **93%** |
| **OES 103 Tumour** | **490.4** | **2.0** | **2.1** | **9.1** | **9%** | **91%** |
| **OES 104 Normal** | **783.2** | **2.1** | **2.1** | **9.4** | **6%** | **94%** |
| **OES 104 Tumour** | **513.6** | **1.9** | **2.1** | **9.3** | **7%** | **93%** |
